# Analyzing Information Seeking and Drug-Safety Alert Response by Health Care Professionals as New Methods for Surveillance

**DOI:** 10.2196/jmir.4427

**Published:** 2015-08-20

**Authors:** Alison Callahan, Igor Pernek, Gregor Stiglic, Jure Leskovec, Howard R Strasberg, Nigam Haresh Shah

**Affiliations:** ^1^ Stanford Center for Biomedical Informatics Research Stanford University Stanford, CA United States; ^2^ Pervasive Computing Applications Research Studios Austria Vienna Austria; ^3^ Faculty of Health Sciences University of Maribor Maribor Slovenia; ^4^ Faculty of Electrical Engineering and Computer Science University of Maribor Maribor Slovenia; ^5^ Computer Science Department Stanford University Stanford, CA United States; ^6^ Wolters Kluwer Health San Diego, CA United States

**Keywords:** Internet log analysis, data mining, physicians, information-seeking behavior, drug safety surveillance

## Abstract

**Background:**

Patterns in general consumer online search logs have been used to monitor health conditions and to predict health-related activities, but the multiple contexts within which consumers perform online searches make significant associations difficult to interpret. Physician information-seeking behavior has typically been analyzed through survey-based approaches and literature reviews. Activity logs from health care professionals using online medical information resources are thus a valuable yet relatively untapped resource for large-scale medical surveillance.

**Objective:**

To analyze health care professionals’ information-seeking behavior and assess the feasibility of measuring drug-safety alert response from the usage logs of an online medical information resource.

**Methods:**

Using two years (2011-2012) of usage logs from UpToDate, we measured the volume of searches related to medical conditions with significant burden in the United States, as well as the seasonal distribution of those searches. We quantified the relationship between searches and resulting page views. Using a large collection of online mainstream media articles and Web log posts we also characterized the uptake of a Food and Drug Administration (FDA) alert via changes in UpToDate search activity compared with general online media activity related to the subject of the alert.

**Results:**

Diseases and symptoms dominate UpToDate searches. Some searches result in page views of only short duration, while others consistently result in longer-than-average page views. The response to an FDA alert for Celexa, characterized by a change in UpToDate search activity, differed considerably from general online media activity. Changes in search activity appeared later and persisted longer in UpToDate logs. The volume of searches and page view durations related to Celexa before the alert also differed from those after the alert.

**Conclusions:**

Understanding the information-seeking behavior associated with online evidence sources can offer insight into the information needs of health professionals and enable large-scale medical surveillance. Our Web log mining approach has the potential to monitor responses to FDA alerts at a national level. Our findings can also inform the design and content of evidence-based medical information resources such as UpToDate.

##  Introduction

Searching and consuming medical information resources on the Web occupies an increasingly important place in both consumer and health care professionals’ day-to-day information-seeking activities [1-6]. Methods for mining Web search logs to characterize user behavior and to perform large-scale surveillance—such as Google Flu Trends [7] and similar efforts using Wikipedia Web traffic [8]—are gaining traction. Studies have sought to characterize the search behavior of specific user groups, for example those who search for information about cancer [9] or varicose vein treatment [10], as well as the effect of significant large-scale events like the recession [11] or the time of year [12] on searches related to health concerns. Patterns in search logs have also been used to predict health-related activities, including visits to medical facilities [13] and the onset of searches about mood-stabilizing drugs [14], as well as to track changes in drug use over time [15].

A challenge inherent in the analysis of Web search behavior generally is the diversity of users. Web search logs capture a broad range of online behavior from a largely uncharacterized user group performing searches with unknown context(s). Most efforts at analyzing Web logs focus on consumer search behavior. In fact, recent methods attempt to discern and separate out searches from health care professionals in the analysis [16].

In contrast, the analysis of search behavior of health care professionals has typically focused on literature review or survey-based approaches [4,17-20]. To directly study information seeking by health care professionals "in the wild" we present an analysis of activity logs from a widely used online medical resource called UpToDate [21]. UpToDate is a source of expert-authored health information provided by Wolters Kluwer that includes detailed descriptions of approaches to investigating specific symptoms, management of diseases, drug usage recommendations, and treatments to support evidence-based medicine. UpToDate is used on a subscription basis by institutions and individuals who purchase a license, including physicians, researchers, and students. Its use in hospitals is known to be associated with fewer patient complications and adverse events, shorter hospital stays, reduced mortality rates, and higher quality performance measures [22,23]. Given the challenges in analyzing general Web search behavior, the UpToDate logs are a unique resource—they capture the search behavior of a limited and well-defined user group. UpToDate logs have been previously used to predict flu trends in a timely manner [24], demonstrating the utility of this resource as an alternative data source for medical surveillance.

Logs of UpToDate usage capture the source institution and a unique deidentified session, the search string entered, the time and date of the search, the type of search, and topic pages visited as a result of a search. Using these access logs, we analyzed both the free-text searches users performed as well as how they navigated UpToDate topic pages. We profiled how UpToDate is used nationally and we quantified the relationship of medical conditions to cost and utilization as seen from an information-seeking perspective. We also characterized information-seeking behavior via the relationship between search terms and subsequent page view duration—a well-studied indicator of user interest [25,26]. We identified patterns in UpToDate page view sequences and the search terms that initiated them. We also present results on the use of UpToDate logs to monitor uptake of a Food and Drug Administration (FDA) alert.

In the following sections, we describe our methods for analyzing the free-text searches and page view sequences from UpToDate search logs. We then present findings of seasonal distributions of UpToDate free-text searches, as well as the distribution of searches by body systems, health conditions and symptoms, drugs, medical devices, and procedures. We also describe the uptake of an FDA drug alert as reflected by changes in the frequency and duration of UpToDate searches and page views. Lastly, we discuss the implications of our findings, describe the limitations of our approach, and propose future work.

## Methods

### Overview

To profile the online search behavior of health care professionals, we used 2 years of usage logs from UpToDate, spanning from January 2011 to December 2012. We analyzed these logs using a combination of text mining and statistical methods to identify general trends in searches, to discover associations between search terms and the duration of subsequent visits to UpToDate topic pages ("topic views") as well as patterns in topic view sequences, and to monitor the uptake of an FDA alert by health care professionals. In the following sections, we describe the structure of the search logs, our text-mining approach, and methods for analyzing UpToDate user behavior as well as identifying patterns in searches and topic views.

### Structure of UpToDate Search Logs

The log for a single UpToDate user event consists of the following parts: (1) query string, (2) unique session ID, (3) search location, (4) time stamp of the search, and (5) the type of action (eg, a string search in the search bar of the website, a topic view, or a subtopic view, which is recorded when a user clicks on a link within an UpToDate page).

We restricted this dataset to consider only searches or page views performed at sites in the United States that purchased an UpToDate license (ie, we have excluded users of trial or marketing versions of UpToDate, as well as those searches performed on computers outside of the United States). The dataset used contains 212 million search queries and their corresponding topic views.

### Text Processing of Search Logs

We processed all free-text searches using a variation of our previously described text-processing workflow [27,28]. We used the Unitex corpus processor to annotate search strings with a lexicon of more than 3 million terms compiled from biomedical ontologies and terminologies, in which terms and concepts are mapped by synonymy and parent-child relationships. The output of this annotation process is a list of terms for each query string, each of which is mapped to one or more biomedical concepts. Concepts in the lexicon are in turn mapped to one of four semantic types, where applicable—diseases and symptoms, drugs, medical devices, and procedures.

### Analyzing Free-Text Searches

Using the output of our text-processing pipeline, we computed the frequency of occurrence of each term across all usage logs, and used these frequencies to profile how health care professionals search UpToDate. We first profiled search behavior using the following features: seasonality, search session length, body system, medical concept category, and national statistics on the costs and discharge rates of medical conditions in 2011 and 2012. We used the time stamp associated with each search to aggregate searches by month and year. We used the concept mappings in our terminology to identify term sets for the 10 major body systems: cardiovascular, respiratory, digestive, endocrine, hemic and immune, integumentary, musculoskeletal, mouth and jaw, nervous, and urogenital. Using these term sets, we counted the number of searches containing any of the terms associated with a given body system. Using the concept mappings in our terminology, we determined the most frequently searched-for disease/symptom, drug, device, and procedure terms, and quantified the distribution of these categories for each body system to assess the comprehensiveness of search logs as a data source.

**Figure 1:**
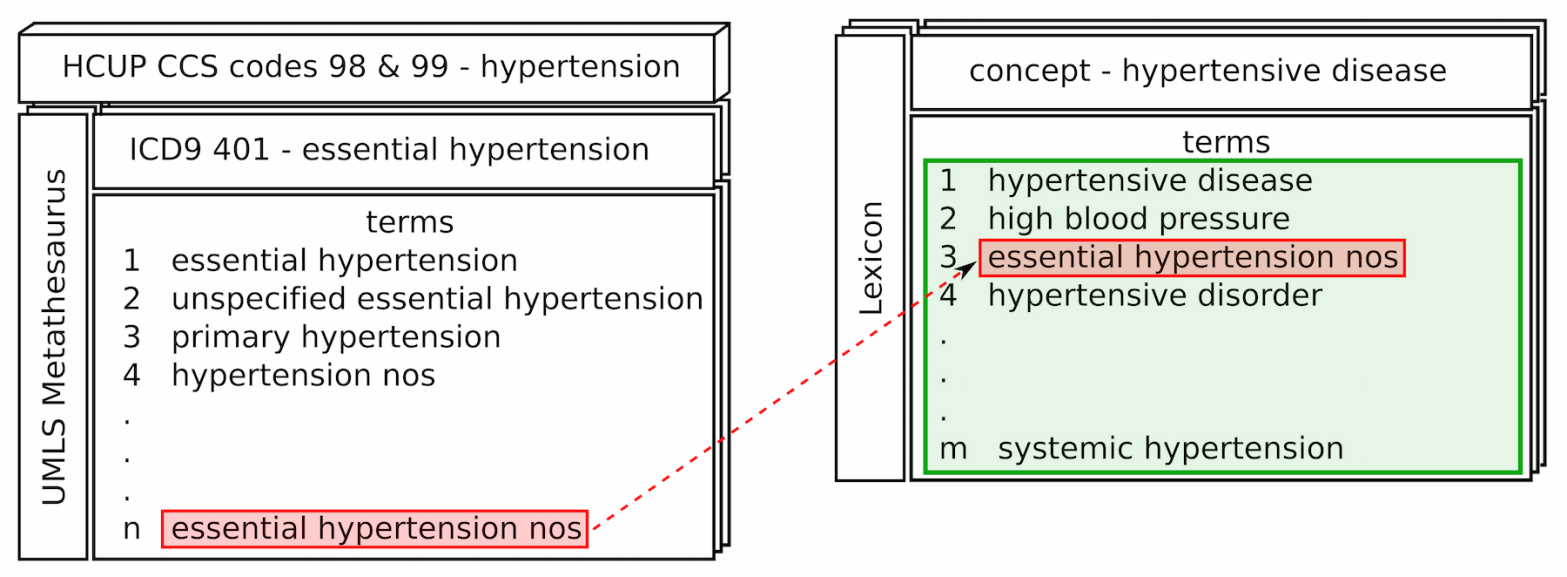
Example of term expansion for HCUP CCS codes; each code is represented as its set of ICD-9 codes. The ICD-9 code for essential hypertension is expanded using the UMLS Metathesaurus to identify the n terms mapped to that concept. Each of the n terms (eg, “essential hypertension nos,” red) is used as a seed query to our custom lexicon to identify additional concepts and their terms. Here, “essential hypertension nos” maps to the concept hypertensive disease, and its additional m-1 terms (green) are used in combination with the n seed terms to identify searches corresponding to the HCUP CCS codes for hypertension.

To explore relationships between the search behavior and national trends of medical conditions in the US, we created custom term sets from our lexicon corresponding to the Healthcare Cost and Utilization Project (HCUP) clinical classification system (CCS) codes. We started with the International Classification of Diseases, 9th Revision (ICD-9) codes that make up each CCS code, and collected the Unified Medical Language System (UMLS) Metathesaurus concepts for each of the ICD-9 codes. We then extended the terms sets associated with these concepts by leveraging the term-concept mappings in our much larger lexicon (see Figure 1), and manually reviewed the terms sets to remove overly broad or incorrect terms. We used these curated term sets to identify searches in UpToDate that are related to medical conditions or procedures for which national data are available from the HCUP National Inpatient Sample.

### Analyzing Information-Seeking Behavior

We used time stamps for all searches and topic views to determine duration of topic views immediately following a given search. Topic view duration was calculated by taking the difference between the time stamp of a topic view event and the time stamp of the next event that followed it—either a search or view of a completely different topic. If the final event within a session was a topic view, that topic view event was excluded from this part of our analysis because it was not possible to calculate the duration of this topic view without the logged time stamp of a subsequent user action. From the log-normalized distribution of time spent on all topic pages following a search, we categorized the amount of time spent on a given topic page using the mean of the log-normalized duration values (converted back to seconds) as the decision boundary. Durations less than this boundary were categorized as *short clicks*, and durations above this boundary were categorized as *long clicks*.

We grouped all searches that initiated a given topic view to calculate the proportion of long clicks and short clicks for each topic, and to determine the number of unique search terms resulting in a topic view. We also grouped all topics resulting from a unique search term to calculate the proportion of long clicks and short clicks originating from the search term and the number of topics each search term initiated.

### Mining Patterns of Topic View Sequences

**Figure 2:**
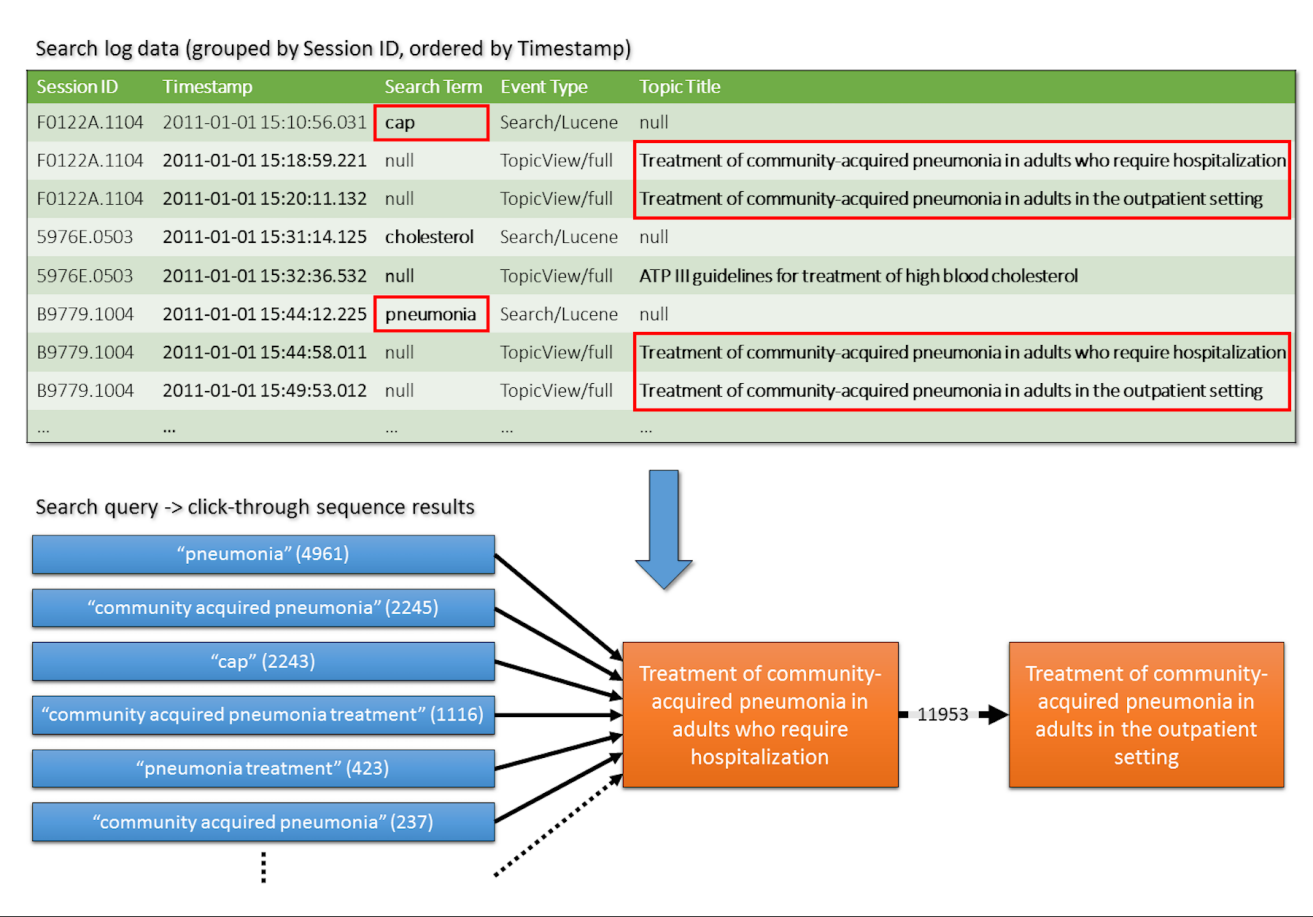
Overview of the method used to analyze sequences of queries and topic views in UpToDate. Using unique session identifiers, we grouped queries and topic views, and ordered them by time of occurrence (green table). Frequently occurring sequences of topic views (orange boxes) were identified, as well as the search terms that initiated them (blue boxes). The numbers in parentheses show the frequency of occurrence of the specific term.

UpToDate content is structured as topics, each of which has a dedicated page with subsections containing more specific information. In addition to analyzing the search text, we used the logs to study how UpToDate users progress from topic to topic within a session, and to profile the search terms that initiate sequences of topic views. Figure 2 summarizes our method. We first grouped searches by their unique session identifier and ordered the entries by the time stamp. For each sequence of two or more topic views, we counted the number of times that sequence occurred, as well as the search terms that occurred just prior to the sequence and their frequency.

### Mining UpToDate Logs to Measure Food and Drug Administration Alert Uptake

On August 24, 2011, the FDA published an alert describing the risk of adverse cardiovascular events associated with higher doses of citalopram (trade name Celexa). We examined the uptake of this alert in the UpToDate logs relative to consumer online media activity from the same time period. We used a large online media collection to compare the relative frequency of terms related to Celexa in the UpToDate search logs and in online news media. The online media collection consisted of over 6 billion online news articles, news wires, and blog posts published on the Web between 2009 and 2014 [29,30]. A single entry included the title, time stamp, URL of the article, as well as the article content. To obtain the documents, we used Spinn3r Web service [31] that monitors over 20 million Internet sources to retrieve approximately 3.2 million new documents each day. The collection represents a near-complete picture of US online media space.

We obtained the daily counts of the mentions of Celexa-related search terms in the news media collection and a cumulative daily count of all Celexa-related search queries on a specific day from the UpToDate logs. Using these counts, we calculated a 7-day moving average of the fraction of total daily search queries that were Celexa related. Similarly, we calculated daily counts and a 7-day moving average of the number of online media articles that contained Celexa-related terms. To enable an overlay of the UpToDate and online news counts, we scaled the UpToDate counts by 10^7^. We considered a deviation from the mean number of occurrences over the 2-year time period as a signal.

## Results

### Overview

UpToDate use was dominated by searches for disease conditions and symptoms and showed significant seasonal variation. The conditions and symptoms that had a high query volume were not those with the greatest burden on the US health care system. For example, headache and viral infections were in the top 20 medical conditions searched for in the study period, but have low relative aggregate cost and discharge rates. We also found that several medical conditions and procedures with high overall expenditures had low query volumes, such as heart attack and mood disorders. The top 1% of institutions by query volume were responsible for about 21% of queries in the United States, and issued an average of 1.7 million queries each. The rest of the queries were widely distributed nationally with some queries coming from each of the fifty states.

We summarized searches and the topic views that followed them in three ways. For topics, we found that topics with longer-than-average dwell times (also known as *long clicks*) had significantly fewer initiating search terms than those with shorter-than-average dwell times (*short clicks*). For search terms, we found that topic views initiated by a given search term were either, on average, longer than the mean topic view duration (ie, that search term always results in long clicks) or were, on average, shorter than the mean topic view duration (ie, that search term always results in short clicks). Finally, we elucidated patterns of frequently occurring search and topic view sequences, which typically started with a search for a disease term and ended with topics related to therapies for that disease.

We then characterized the "response" of the health professionals to a 2011 FDA drug alert—about the risk of abnormal heart rhythms when taking high doses of citalopram (Celexa)—in terms of the change in information-seeking behavior. Our comparison of the volume of searches about citalopram (Celexa) in UpToDate versus mentions of these terms in news sites following a 2011 FDA alert found that the uptake of alerts by health care professionals in their daily work differed markedly from that of the general public. Searches in UpToDate related to citalopram peaked more than 10 days after the FDA alert and the initial spike in mentions at news sites, but persisted for much longer.

### Seasonal and Topic Trends in UpToDate User Search Behavior

#### Seasonal Distribution of UpToDate Searches

**Figure 3:**
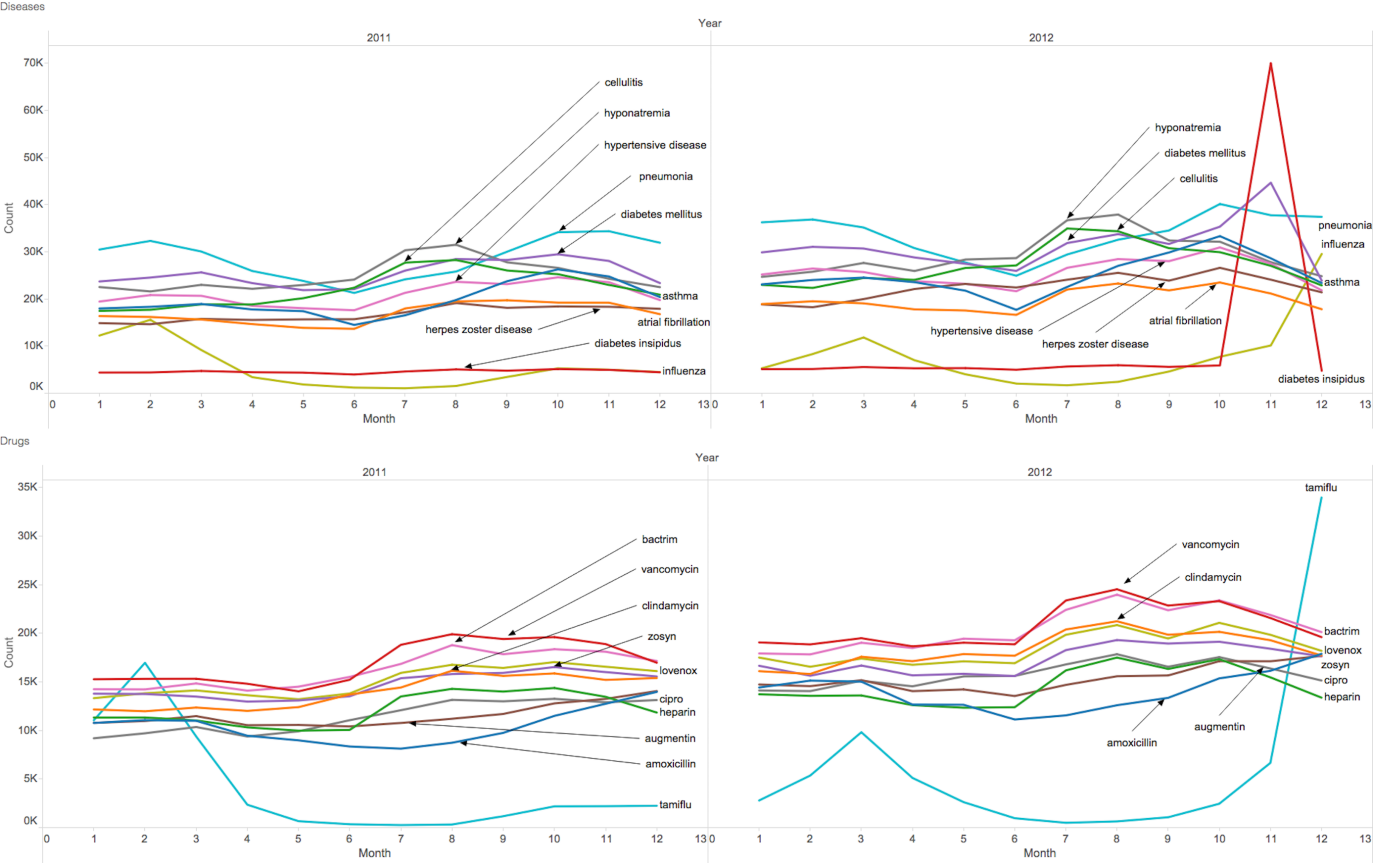
Most frequently searched diseases (top) and drugs (bottom) in 2011-2012.

We measured the monthly frequency of the 10 most searched diseases and drugs in any month of 2011 and 2012 (see Figure 3). There were similar spikes in searches for influenza and Tamiflu (the trade name for oseltamivir, a flu medication) in the early winter of 2011, and winter to early spring of 2012. Searches for pneumonia also peaked in the winter months of both years. There was a sharp increase in the number of searches for diabetes insipidus in November of 2012, representing the disease with the highest search volume within a single month. The most searched-for drugs were vancomycin and Bactrim (the trade name for trimethoprim/sulfamethoxazole), antibiotics that are used to treat a variety of bacterial infections.

#### Distribution of Searches Across Organ Systems

**Figure 4:**
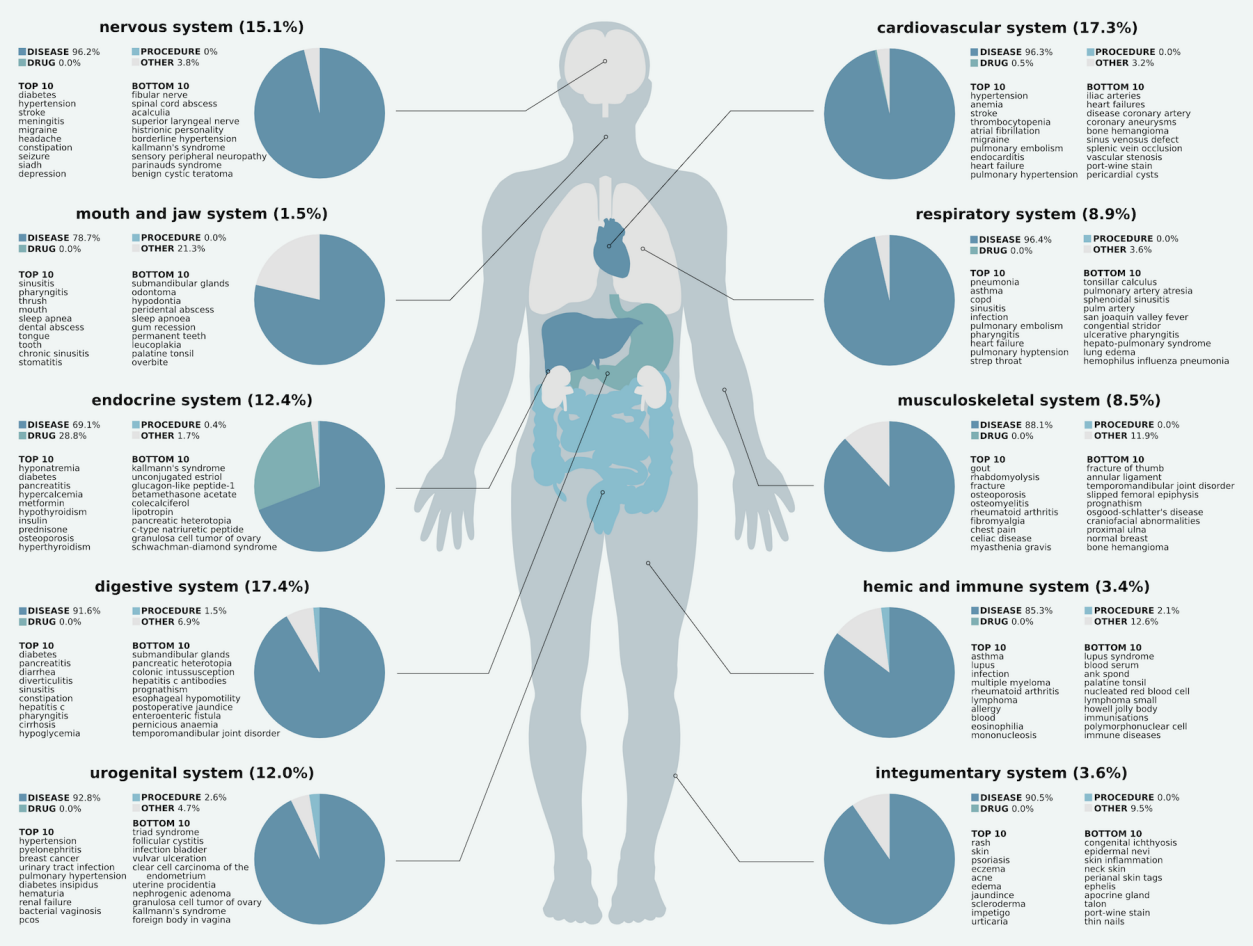
Distribution of searches across organ systems. Each organ system name is followed by the percentage of queries related to that system. Each pie chart shows the distribution of searches for that organ system grouped by their term category, followed by the 10 most frequent and 10 least frequent terms searched for related to that system. Searches about diseases and symptoms (dark blue) dominated most systems. Searches about the endocrine system included a significant number of drug searches, followed by the cardiovascular system.

Figure 4 shows the distribution by type of queries about each of the 10 major organ systems. The digestive system had the highest percentage of searches, followed closely by the cardiovascular system, while the mouth and jaw system had the lowest percentage of searches. Queries about all body systems were dominated by searches for diseases and symptoms. Searches about the endocrine system included a greater percentage of drugs. In general, queries related to medical procedures were much lower in percentage, and were most represented in queries about the hemic and immune system, digestive system, and urogenital system. Figure 4 also lists the 10 most frequently and least frequently occurring terms related to each organ system (note that the categories are not exclusive, because the same term may be related to multiple systems). Characterizing the relative search volume related to the major organ systems is important for assessing the feasibility of using the UpToDate search logs for large-scale surveillance.

#### Distribution of Searches Across Medical Conditions

**Figure 5:**
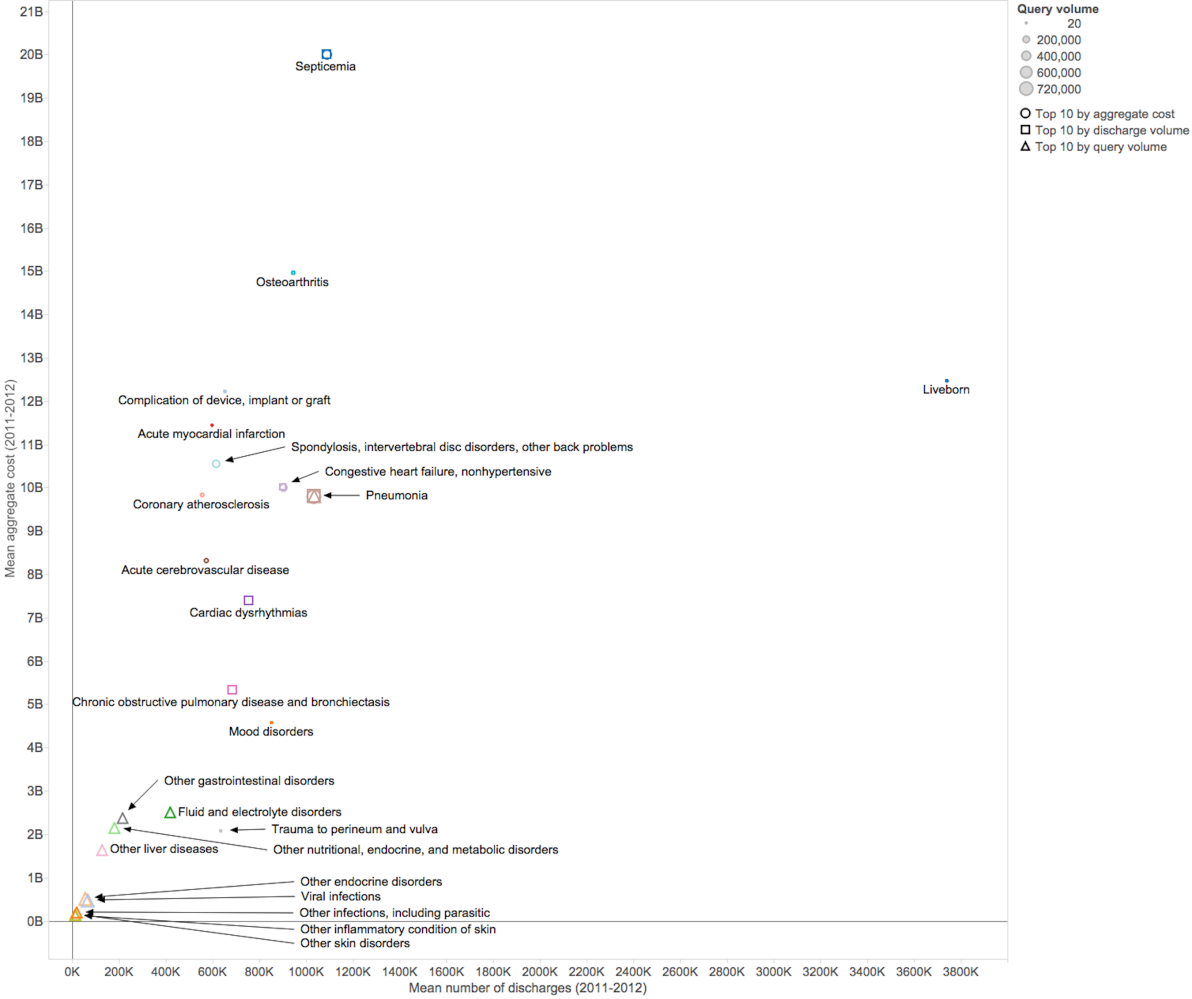
Medical conditions with the highest mean aggregate cost, discharge volume, and/or UpToDate query volume over 2011 and 2012. Each point is a single medical condition and the size of the point represents the query volume. The shape of the point indicates if it was ranked in the top 10 by aggregate cost, discharge volume, and/or query volume. Cost and discharge data were taken from the HCUP National Inpatient Sample.

The query volume for the medical conditions with the most significant burden on the US health care system (quantified as being ranked within the top 10 mean aggregate costs and/or discharge volumes in 2011-2012) varied significantly across conditions (see Figure 5). Some of these medical conditions had very low query volumes in UpToDate—for example, live births, heart attack, and mood disorders—while others, such as septicemia and pneumonia, were frequently searched for. It is not surprising that conditions such as live birth or heart attack have low query volumes—these are medical events that have well-understood management protocols, and thus it is expected that there is relatively little information need associated with them.

The most-searched medical conditions (the triangles in Figure 5) ranged from low aggregate cost and low discharge volume to high aggregate cost. Inflammatory skin conditions and infections had high query volume, but low aggregate cost and number of discharges. Septicemia and pneumonia were high on all three axes of cost, discharge, and query volume. These conditions with high query volume and burden represent surveillance opportunities for both public health and drug safety.

### UpToDate User Behavior

**Figure 6:**
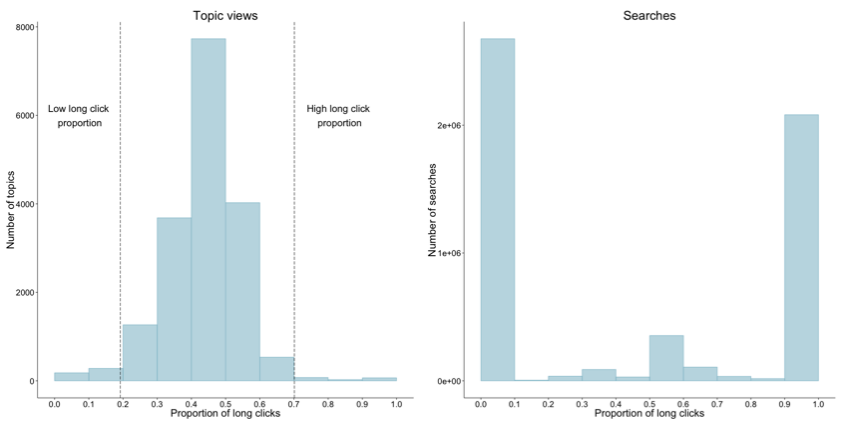
Distribution of long clicks in UpToDate topics (left) and search terms (right). The dotted lines in the left panel indicate the threshold for low and high long-click proportions determined using the Hampel identifier for outliers.

To characterize how users search and consume UpToDate content, we analyzed the relationship between searches that users performed and the amount of time spent looking at the UpToDate topics returned as results for those searches. As described in the Methods section, we used the distribution of topic view durations to decide the cutoff duration (143.79 seconds) to categorize each topic view as a *short click* or a *long click*. We then determined the proportion of long clicks for each topic. The left panel of Figure 6 shows the distribution of long-click proportion across topics. We used the Hampel identifier for outliers to determine the threshold for high (0.70) and low (0.19) proportions of long clicks (shown as dotted lines in the left panel of Figure 6).

Topics with a high proportion of long clicks originated from significantly fewer unique searches than topics with mostly short clicks. Topics with a high proportion of long clicks had an average of 10.37 unique initiating search terms (SD 22.13) and a median of 4 (interquartile range [IQR] 2) unique initiating search terms. This is significantly lower than the average of 29.29 (SD 50.22) and median of 12 (IQR 8) unique initiating search terms for topics with a high proportion of short clicks (*P*<.001; Mann-Whitney U test).

In contrast, the distribution of long-click proportion for searches (the proportion of topic views with a long click initiated by a given search term) was distinctly bimodal (right panel of Figure 6). Searches tended to have either no long clicks following them, or mostly long clicks following them. This indicates that while topics typically had a number of search terms that landed the user on that topic and kept them reading for a long period of time, a given search term either always resulted in long clicks or always resulted in short clicks. Table 1 lists the top 10 search terms (by frequency in the logs) that always resulted in short clicks. Search terms that always result in short clicks may be terms that are too specific to return useful content or contain a typographical error (eg, "phenochromocytoma" and "probencid" are misspellings of the disease pheochromocytoma and the drug probenecid, respectively), but some may be candidates for addition to UpToDate content. Table 2 lists the top 10 search terms (by frequency in the logs) that always resulted in long clicks.

**Table 1:** Top 10 search terms (by volume) that did not initiate any long clicks for subsequent topic views.

Term	Frequency
gad7	187
adriamycin patient info	144
doxorubin patient info	109
phenochromocytoma	106
lab values in pregnancy	105
augment	86
probencid	79
dilaudid in pregnancy	71
parovirus	66
kidney ston	60

**Table 2:** Top 10 search terms (by volume) that initiated only long clicks for subsequent topic views.

Term	Frequency
abreva	3411
bullous myringitis	3392
subchorionic hemorrhage	1904
choroid plexus cyst	1270
cerefolin	1258
estropipate	1207
moexipril	1181
postpartum hypertension	1153
tinactin	1152
flucon	1114

### Sequences of Searches and Topic Views

Table 3 lists the most frequently observed topic sequences of lengths 3 and 4, and the searches that initiated them. We found that topic sequences longer than 4 had a high proportion of topic "switching" (alternating repeatedly between two topics) and are thus not shown. These topic sequences had a common pattern: a topic view for a disease or condition concept was followed by topic views for drugs or treatments for the disease.

**Table 3:** The 10 most frequently observed topic view sequences of size 3 or 4, and the search terms that initiated them.

Topic view sequence (frequency)	Initiating search terms (frequency)
Cellulitis and erysipelas → Clindamycin: drug information → Clindamycin (systemic): drug information (14,463)	cellulitis (10,434), cellulitis treatment (2055), erysipelas (260), skin infection (219), facial cellulitis (171)
Onychomycosis → Terbinafine: drug information → Terbinafine (systemic): drug information (8234)	onychomycosis (3421), onychomycosis treatment (1433), onchomycosis (531), toenail fungus (524), nail fungus (476)
Onjunctivitis → Erythromycin: drug information → Erythromycin (ophthalmic): drug information (5938)	conjunctivitis (2738), conjunctivitis treatment (801), pink eye (603), bacterial conjunctivitis (422), conjunctivitis (292)
Treatment of clostridium difficile infection in adults → Metronidazole: drug information → Metronidazole (systemic): drug information (5923)	c diff (1947), c diff treatment (454), clostridium difficile treatment (439), clostridium difficile (416), c. diff (357)
Treatment of acute pancreatitis → Predicting the severity of acute pancreatitis → Calculator: Ranson criteria for pancreatitis prognosis → Calculator: Apache II scoring system (1219)	pancreatitis (731), acute pancreatitis (303), pancreatitis treatment (73), acute pancreatitis treatment (52), gallstone pancreatitis (23)
Management of acute exacerbations of chronic obstructive pulmonary disease → Management of infection in acute exacerbations of chronic obstructive pulmonary disease → Azithromycin: drug information → Azithromycin (systemic): drug information (565)	copd exacerbation (350), copd (98), copd exacerbation treatment (80), copd exacerbation antibiotics (24), copd exac (8)
Overview of diaper dermatitis in infants and children → Nystatin: drug information → Nystatin (topical): drug information → Nystatin (topical): pediatric drug information (522)	diaper rash (350), diaper dermatitis (71), diaper rash treatment (36), diaper candidiasis (19), candidal diaper rash (18)
Acute uncomplicated cystitis and pyelonephritis in women → Ciprofloxacin: drug information → Ciprofloxacin (ophthalmic): drug information → Ciprofloxacin (systemic): drug information (416)	uti (276), urinary tract infection (52), cystitis (31), uti treatment (26), pyelonephritis (16)
Overview of acute pulmonary embolism → Diagnosis of acute pulmonary embolism → Treatment of acute pulmonary embolism → Anticoagulation in acute pulmonary embolism (392)	pulmonary embolism (311), pe (50), pulmonary embolus (31)

### Monitoring Response to a Food and Drug Administration Alert Using UpToDate and Online Media Activity

**Figure 7:**
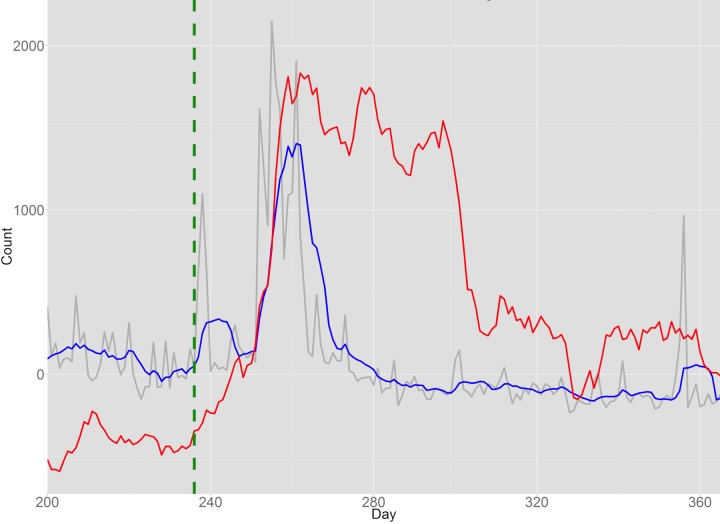
Scaled 7-day moving average of UpToDate query volume (red), 7-day moving average of media activity (blue), and raw media activity volume (grey) related to Celexa prior to and following the August 24, 2011 FDA warning (date indicated by the green dotted line).

Finally, we used user search activity as seen in UpToDate logs to monitor the response of health care professionals to FDA alerts. Specifically, we measured the relative volume of searches about the antidepressant drug citalopram (trade name Celexa) prior to and following the August 24, 2011 FDA warning about the risk of abnormal heart rhythms when using citalopram. As described in the Methods, we compared the relative query volume in UpToDate to the relative volume of mentions for the drug in online media (see Figure 7 and Multimedia Appendix 1).

Online media showed a small surge in Celexa-related terms on the same day as the alert, and showed a sharp rise in the following days peaking at approximately 10 days. In contrast, the UpToDate query volume for Celexa did not increase until 10 days *after* the FDA alert but had sustained high query volumes for approximately the next 60 days, long after Celexa-related activity in the general online media returned to a baseline level.

The number of unique search terms that initiated Celexa-related topic views was much higher after the alert (see Table 4), but the proportion of long clicks for those topics was lower after the alert. This difference was even more pronounced when considering only the 2 months prior to and after the alert (data not shown). The average topic view duration for Celexa pages was also significantly lower after the FDA alert (842.31 seconds in the ~9 months before the alert compared to 744.36 seconds in the ~9 months after the alert).

Search specificity also increased following the FDA alert. The number of searches for “citalopram” or “Celexa” with terms “long qt,” “heart,” or “rhythm” was only 2 before the alert and 34 after the alert, demonstrating the effect of the FDA alert.

**Table 4:** Number of unique search terms that initiated Celexa (citalopram) topic views, and the proportion of long clicks for those topics before and after the August 24, 2011 FDA warning.

Topic	Number of unique search terms		Proportion of long clicks	
	Before alert	After alert	Before alert	After alert
Citalopram: Drug information	489	683	0.376	0.354
Citalopram: Patient drug information	183	303	0.262	0.247
Citalopram: Pediatric drug information	43	63	0.395	0.301

## Discussion

### Principal Findings

This is the first study profiling the online search behavior of medical professionals using a dedicated evidence-based medical information resource. Our findings can inform changes in the design of resources such as UpToDate. For example, search terms that only resulted in short clicks (indicating that the results were not useful to the searcher) potentially identify the need for new content or new search terms, while search terms and topics that have only high proportions of long clicks represent well-covered topics. Searches that have a seasonal variation in volume, such as searches for influenza and related medications, could be highlighted in the appropriate season to make the content more accessible. Such analyses can improve the utilization of the content by taking into account user behavior—an activity that consumer Web companies routinely undertake.

Recent work on mining Web logs in the medical domain has been concerned with pharmacovigilance using consumer logs [13,16,32]. These studies use statistical methods to detect significant single- and multi-drug adverse event association signals from consumer search logs, taking into consideration the length of time between searches for drugs and adverse events and using known drug-induced adverse events as gold standards to evaluate the performance of their methods. For example, an analysis of consumer search logs showed that evidence of a multi-drug adverse event association between hyperglycemia, pravastatin, and paroxetine could be detected by quantifying the disproportionality of searches about hyperglycemia occurring with searches about both pravastatin and paroxetine, as compared to its co-occurrence with searches for only one of the drugs [32]. A second study demonstrated that combining data from the FDA Adverse Event Reporting System (FAERS) with consumer search logs improved accuracy of adverse drug event detection by 19%, compared with using either source alone [16]. Our findings in this work and in a recent preliminary study [33] indicate that incorporating search logs of health care professionals for pharmacovigilance is a promising approach.

The uptake of the 2011 FDA alert for citalopram was clearly reflected in UpToDate logs, demonstrating the potential of this data source as a means to assess the efficacy and measure uptake of FDA alerts by health care professionals. This finding is also supported by previous work demonstrating the analysis of UpToDate usage logs to monitor influenza epidemics [24]. In combination with a recently published time-indexed reference set of adverse drug reactions extracted from recent FDA label changes and warnings [34], UpToDate logs could enable a large-scale analysis of physician response to FDA label changes and alerts. Our data extract (from 2011-2012) does not overlap with the time-indexed label changes. However, with access to recent data, such an analysis has the potential to inform the FDA of the effectiveness of their alerts more broadly.

Search log analysis may offer additional opportunities for surveillance by measuring changes in search volume about organ systems, diseases, and drugs over time, as well as by quantifying the relationship between changes in search volume and associated events. Such surveillance can monitor infectious disease outbreaks or watch for changes in the prevalence of health conditions that are a significant burden on the health care system. Development of methods to monitor such changes is possible and has been previously demonstrated by the use of consumer Internet search logs to predict health care utilization, detect flu outbreaks, and to track prescription drug use. The use of health professional search logs to improve upon such use cases is a research area with the potential to improve public health through earlier warning of disease outbreaks and to improve drug safety surveillance by monitoring physician response to FDA communications to assess their efficacy.

### Limitations

There are several limitations to our approach. While the location, time, and associated user license of UpToDate searches and topic views are known, we do not have information on the identity of UpToDate users. We expect that the vast majority of licensed UpToDate users are health care professionals (ie, medical doctors, nurse practitioners, and/or researchers), but it is possible that some of the logs capture UpToDate use by patients or other types of consumers. Similarly, a unique session identifier relates searches and topic page views, but it is possible that within a single session there were multiple users with distinct information needs and behavior. Relying on the raw logs, we are unable to identify user switching within sessions, and as a result may have associated searches and topic views that were actually performed by different users and are therefore unrelated. However, research has been dedicated to developing methods for automatically determining session boundaries in Web log data (including user switching)*—*for example, in work by Göker and He [35] and Murray et al [36]—which may be used to address this potential shortcoming. Also, as noted in the Methods, we ignored topic view events that were the final event in a session because it was not possible to calculate page view duration for those topic views. This reduced the number of topic view events available for analysis by 22%; the remaining 78% of topic views spanned 63% of all sessions. Finally, our findings are based on a relatively short surveillance period of 2 years—an analysis of logs from a longer period could reveal new associations or associations of differing strength.

More generally, data mining approaches applied to Web-scale search data may have methodological shortcomings. In early 2013 it was found that the Google Flu Trends system was overestimating flu prevalence, predicting values much higher than estimates from the Centers for Disease Control. Such inaccuracies may occur when methods are not recalibrated to adjust for temporal fluctuations that have an external cause, such as media coverage of the unusual 2012-2013 flu season resulting in more flu-related searches [37]. An analysis of Google Flu Trends data found that its prediction errors from week to week were correlated to each other and exhibited seasonality [38], suggesting that additional confounding variables (including changes to Google search algorithms themselves) may be partly responsible for changes in observed search term prevalence. Such potential confounders should be considered when interpreting our FDA alert findings, but could not be included in our analysis because we do not have access to UpToDate search engine features or ranking algorithms.

### Conclusions

Our results demonstrate that mining UpToDate search logs offers unique insight into the information-seeking behavior of health care professionals, and the relationship between this behavior and health care utilization associated with disease states. Our results allow us to understand the information needs of health professionals in their day-to-day practice and the relationship between search terms and topic views—a large fraction of which include a disease or condition concept followed by drugs or treatments for the disease. Lastly, we were able to use UpToDate to quantify the uptake of the FDA alert for a serious drug adverse event, illustrating a novel use of analyzing search behavior to monitor responses to FDA alerts at a national level.

## Appendix

Multimedia Appendix 1 Scaled 7-day moving average of UpToDate query volume (red), 7-day moving average of media activity (blue), and raw media activity volume (grey) related to Celexa for all of 2011-2012, prior to and following the August 24, 2011 FDA warning (date indicated by the green dotted line).

## Abbreviations

CCS: clinical classification system
FAERS: Food and Drug Administration Adverse Event Reporting System
FDA: Food and Drug Administration
HCUP: Healthcare Cost and Utilization Project
ICD-9: International Classification of Diseases, 9th Revision
IQR: interquartile range
NIGMS: National Institute of General Medical Sciences
NIH: National Institutes of Health
NLM: National Library of Medicine
UMLS: Unified Medical Language System
