# Supplementary figures and images for: Analyzing Information Seeking and Drug-Safety Alert Response by Health Care Professionals as New Methods for Surveillance
Source: J Med Internet Res. 2015 Aug 20;17(8):e204. doi: 10.2196/jmir.4427 (PMC4642796; doi:10.2196/jmir.4427)

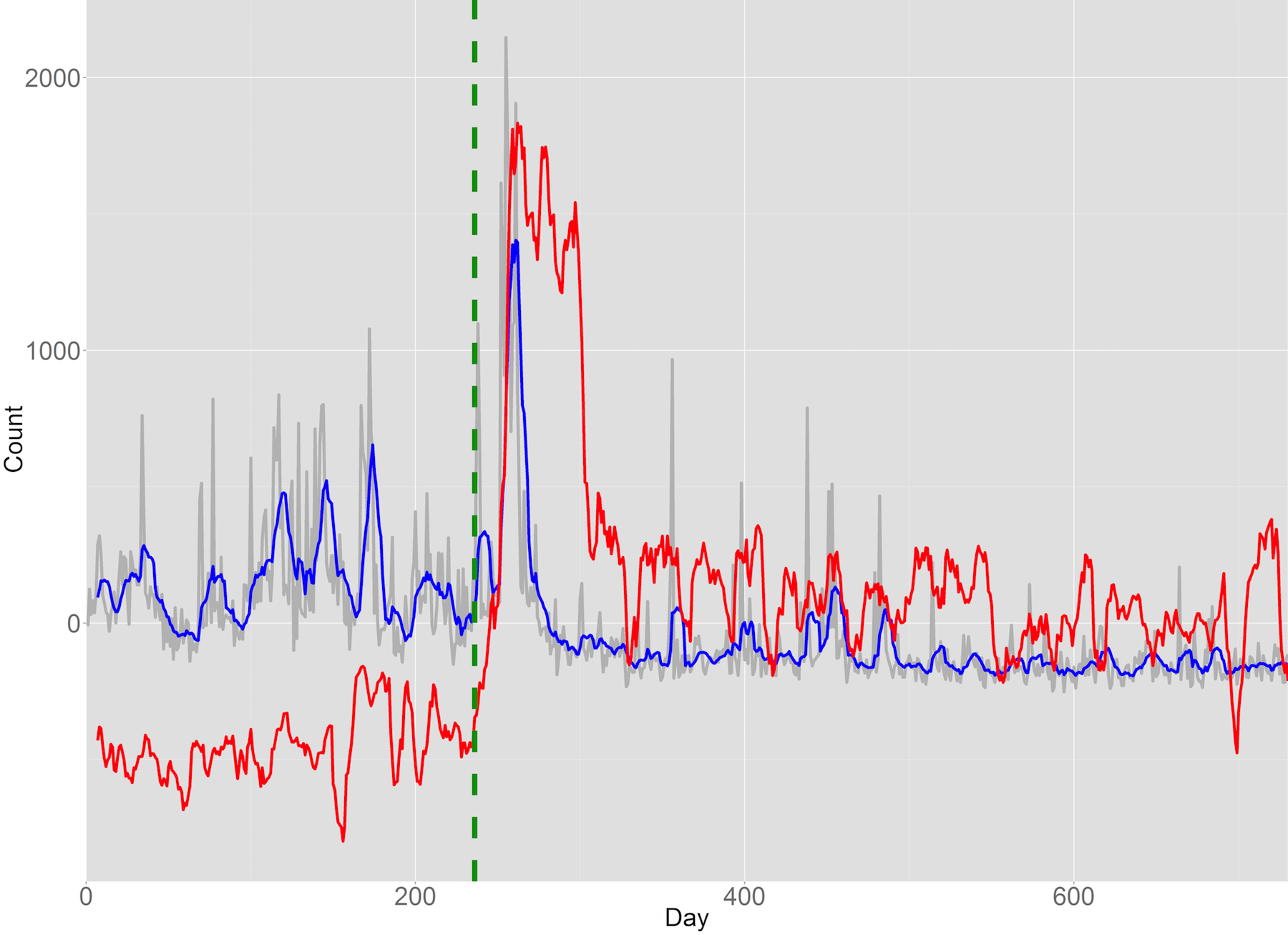

Supplement: Multimedia Appendix 1 [file jmir_v17i8e204_app1.png]
